# Perihippocampal failure after hippocampal-avoidance whole-brain radiotherapy in cancer patients with brain metastases: Results of a retrospective analysis

**DOI:** 10.1097/MD.0000000000029144

**Published:** 2022-04-08

**Authors:** Li-Tsun Shieh, Sung-Wei Lee, Chia-Chun Chen, Yi-Chia Ho, Yu-Wen Wang, Sheng-Yow Ho

**Affiliations:** a Department of Radiation Oncology, Chi Mei Medical Center, Liouying, Tainan, Taiwan; b Department of Internal Medicine, National Taiwan University Hospital, Hsin-Chu Branch, Hsin-Chu, Taiwan; c Department of Radiation Oncology, Ditmanson Medical Foundation Chia-Yi Christian Hospital, Chia-Yi City, Taiwan; d Department of Radiation Oncology, Chi Mei Medical Center, Tainan, Taiwan; e Graduate Institute of Medical Sciences, Chang Jung Christian University, Tainan, Taiwan.

**Keywords:** brain metastasis, hippocampal-avoidance, lung cancer, perihippocampal failure, whole-brain radiation therapy

## Abstract

Perihippocampal failure is a rare clinical scenario in brain metastatic cancer patients following hippocampal-avoidance (HA) whole-brain radiotherapy (HA-WBRT). The clinical features have not been fully identified because clinical data on intracranial failure after HA-WBRT are limited. It is thus necessary to accumulate clinical data.

We retrospectively analyzed cancer patients with brain metastases who were diagnosed between January 2014 and September 2020 at a regional referral hospital. The medical records of patients who underwent HA-WBRT were reviewed. The clinical features of intracranial recurrence were described. Dosimetry parameters were compared in terms of deviation from the recommended protocol of the Radiation Therapy Oncology Report 0933.

Twenty-four eligible patients with brain metastases who underwent HA-WBRT were identified; 13 (54%) were male. Seventeen patients (71%) had lung cancer, 6 (25%) had breast cancer, and 1 (4%) had liver cancer. The median overall survival was 12 months. Three patients developed intracranial failure during clinical follow-up, and 2 relapsed with intracranial failure in the perihippocampal region at 13 and 22 months, respectively. The perihippocampal failure rate was about 8%. One patient with small cell lung cancer received HA-prophylactic cranial irradiation; the minimum and maximum doses to the hippocampi were 6.8 and 10.7 Gy, respectively. Another patient with brain metastases from lung adenocarcinoma received HA-WBRT; the minimum and maximum doses to the hippocampi were 5.4 and 10.6 Gy, respectively.

We reported unusual cases of intracranial failure in the perihippocampal region following HA-WBRT. Perihippocampal failure could be attributed to an under-dose of radiation partially or be resulted from aggressiveness of cancer per se. Further research on this topic is encouraged.

## 1 Introduction

Brain metastases are the most common intracranial tumors in adults. They represent an important cause of morbidity and mortality, occurring in approximately 10% to 30% of adult cancer patients during cancer therapy.^[1]^ The incidence of brain metastases has risen in recent decades, in the context of an aging population, better treatment of systemic disease, and improved imaging methods for detecting smaller brain metastases in asymptomatic patients. Whole-brain radiotherapy (WBRT) has served as the standard palliative treatment for patients with multiple brain metastases. The appropriate use of WBRT can provide rapid attenuation of neurologic symptoms, improve quality of life, and be especially beneficial for patients whose brain metastases are surgically inaccessible or when the patient cannot undergo neurosurgery.^[1–3]^ Until the 21^st^ century, the prognosis of patients with brain metastases was poor. The overall survival of treated patients was approximately 4 to 6 months; long-term cognitive deficits following WBRT were not observed. With the advent of targeted therapies and advanced treatments, cancer patients with brain metastases can now survive longer, even for years following WBRT.^[3]^ Although control of metastatic brain lesions is the most important benefit of WBRT, decline in neurocognitive functions can occur in patients with longer survival times.^[3–5]^

Injury to the neural stem cell compartment has been hypothesized to be central to the pathogenesis of radiation-induced cognitive decline. The neural stem cell compartment, located in the subgranular zone of the hippocampal dentate gyrus, has been associated with the formation of new memory.^[4–5]^ Hippocampal neural stem cell injury during WBRT may play a crucial role in neurocognitive decline. Hippocampal-avoidance (HA)-WBRT poses an important technical challenge with respect to contouring and treatment planning to spare the hippocampus, which is centrally located in the brain. With the advancement of linear accelerator (LINAC)-based intensity-modulated radiotherapy (IMRT) and helical Tomotherapy, highly complex and time-consuming techniques of HA-WBRT treatment have become possible. The Radiation Therapy Oncology Group (RTOG) 0933 trial aimed to investigate the mitigation of radiation-induced neurocognitive toxicity in WBRT.^[6–9]^ Serial studies have reported that HA-WBRT for cancer patients with brain metastasis are associated with improved quality of life and preservation of memory.^[5–10]^

The risk of developing perihippocampal failure after HA-WBRT is unknown.^[6,7]^ Ghia et al reviewed historical medical image datasets of 272 cases with brain metastases, and estimated that the incidence of metastases within 5 mm of the perihippocampal region was low (3.3%).^[11]^

RTOG 0933 recommends stringent dose criteria; it requires a high level of dose homogeneity and precise radiation delivery. In terms of hippocampal dose reduction, planning brain coverage, and homogeneity, planning optimization is very difficult in routine radiotherapy, since both hippocampi are completely surrounded by the planning target volume (PTV). HA-WBRT treatment could come at the cost of compliance with conformity and dose distribution targets in LINAC-based radiotherapy or helical Tomotherapy.^[6–9,12–18]^ However, in clinical practice, inhomogeneous coverage of the brain PTV poses a risk of perihippocampal failure, given possible occult metastatic lesions around the HA region. This presents a dilemma in clinical judgment.

Perihippocampal failure is a rare clinical scenario in brain metastasis following HA-WBRT.^[19–20]^ Its clinical features have not been fully elucidated due to limited clinical data. Observation of a range of clinical cases is required. Herein, we retrospectively analyze perihippocampal failure in brain metastatic cancer patients after HA-WBRT, and assess the dosimetry parameters of HA-WBRT.

## 2 Methods

### 2.1 Study population

We retrospectively analyzed cancer patients with brain metastases who were diagnosed between January 2014 and September 2020 at a regional referral medical center in Tainan, Taiwan. We reviewed the medical records of patients who received HA-WBRT and had available follow-up information. We excluded patients who had previously received brain irradiation, or who did not complete HA-WBRT. Patients eligible for selection to receive WA-WBRT were older than 20 years of age with a fair-to-good performance status and Eastern Cooperative Oncology Group score of ≤2. Brain magnetic resonance imaging (MRI) was arranged to exclude >4 metastatic foci, tumor >4 cm in diameter, or metastasis within 5 mm perihippocampally. We extracted the following information from the medical database: age, sex, histology, radiotherapy plan, dosimetry information, recurrence, and date of last follow-up. The follow-up time was from the date of detection of brain metastases to December 2020. The study protocol was approved by the Institutional Review Board at our institution (IRB 10603-L06).

### 2.2 Treatment planning and delivery

All enrolled patients underwent a computed tomography simulation scan encompassing the entire head region, with 2-mm slice thickness, using a thermoplastic mask for immobilization. All patients should have had brain MRI before HA-WBRT so as to delineate the bilateral hippocampus; the delineation was established and confirmed by an experienced radiation oncologist. HA regions will be generated by 3-dimensionally expanding the hippocampal contours by 5 mm to allow for the sharp dose fall-off between the bilateral hippocampal structures. The clinical tumor volume (CTV) is defined as the whole-brain parenchyma. The PTV is defined as the CTV minus the HA regions. Treatment plans were generated for 6-MV photons beams using the Pinnacle (Philips, Fitchburg, WI) and TomoTherapy (Accuray, Sunnyvale, CA) planning systems. The detailed planning technique was reported in our previous study (14). The prescribed dose of prophylactic cranial irradiation (PCI) was 25 to 27 Gy in 10 to 15 fractions in patients with small cell lung cancer (SCLC), or 30 Gy in 10 to 12 fractions for brain metastases. An additional boost to gross lesions was allowed. Follow-up brain MRI was arranged at 3-month intervals after HA-WBRT or shorter interval upon recurrent symptoms of brain metastasis including headaches, seizures, or detectable changes in mental or motor function.

### 2.3 Dosimetry analysis of HA-WBRT

The RTOG 0933 compliance criteria for HA-WBRT (target and normal tissue planning doses) are as follows: at least 95% of the brain volume (PTV) receives 30 Gy (V_30Gy_ > 95% PTV), 2% of the target volume receives 37.5 Gy or less (D_2%_ ≤ 37.5 Gy), 98% of the target volume receives 25 Gy or more (D_98%_ PTV ≥25 Gy), minimum dose to the hippocampi (D_min_ = D_100%_) was 10 Gy, maximum dose to the hippocampi was 17 Gy, and maximum dose to optic nerve or chiasm was 37.5 Gy (6, 8). Deviation from the HA-WBRT plan was defined as when the dose parameters are in the unacceptable deviation column of the protocol. Unacceptable deviations include PTV V_30Gy_ < 90%, D_2%_ > 40Gy, hippocampus D_max_ > 17 Gy, D_min_ > 10 Gy, and maximum dose of optic nerve or chiasm >37.5 Gy (6, 8).

### 2.4 Statistical analysis

Descriptive analysis of the clinical features and dosimetry parameters was described. Patients were censored at death or at the date of last follow-up. Overall survival curves were plotted using the Kaplan–Meier method. All analyses were performed using SPSS (Version 24.0. Armonk, NY: IBM Corp). A 2-tailed significance level of 0.05 was set.

## 3 Results

**Figure 1:**
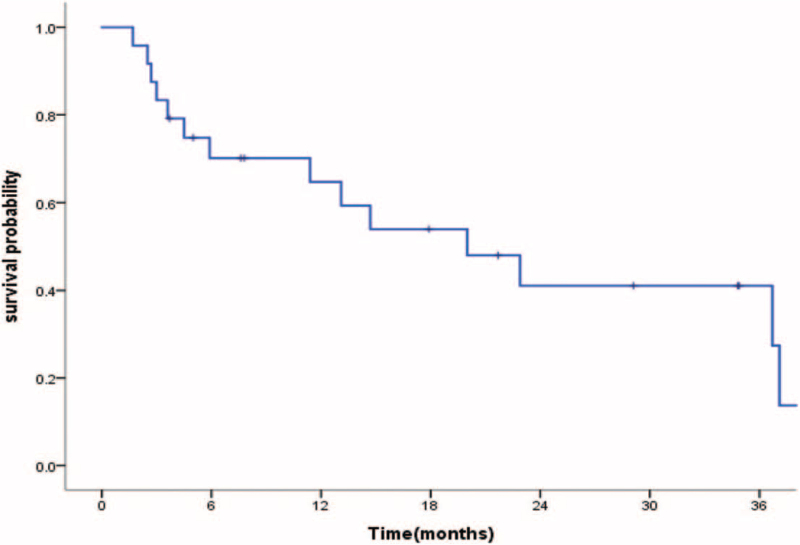
Kaplan–Meir curves of the overall survival of cancer patients with brain metastases who underwent hippocampal-avoidance whole-brain radiotherapy.

Twenty-four eligible patients with brain metastasis who underwent HA-WBRT or PCI were retrospectively identified. The demographic and clinical data of the patients are shown in Table 1. The patients’ ages ranged from 36 to 81 years (median, 59.5 years) at the date of referral for brain irradiation. Thirteen patients (54%) were male. Seventeen patients (71%) had lung cancer, 6 (25%) had breast cancer, and 1 (4%) had liver cancer. In total, there were 50 brain metastatic lesions. The median number of metastases was 2 (range, 0–4). Nineteen patients (79%) underwent LINAC-based treatment and 5 received Tomotherapy. No patient underwent upfront neurosurgical resection. There were 4 SCLC patients, of whom 3 SCLC patients were referred to undergo HA-PCI and 1 patient with brain metastasis to accept HA-WBRT. Regarding extracranial disease, it was clinically controlled in 16 patients (67%). Thirteen patients (54%) received cytotoxic chemotherapy, and 8 (33%) received targeted therapy after brain irradiation. For all patients, the median overall survival was 12 months (368 days). Kaplan–Meir survival curves are shown in Figure 1.

**Table 1:** Demographics and tumor characteristics of brain metastatic patients that received HA-WBRT.

Characteristics	Patients (N = 24) (n, %)
Age, y (median, range)	59.5 (36–81)
Sex	
Man	13 (54)
Woman	11 (46)
Performance (ECOG)	
0–1	17 (71)
2	7 (29)
Histologic type of primary tumor	
Lung, NSCLC	13 (54)
Lung, SCLC	4 (17)
Breast	6 (25)
Liver	1 (4)
No. of brain metastasis at diagnosis	
0	3 (13)
1	8 (33)
2	4 (17)
3	2 (8)
4	7 (29)
Status of extracranial metastasis	
Controlled	16 (67)
Not controlled	8 (33)
Neurosurgery before radiotherapy	
Yes	0 (0)
No	24 (100)
Role of HA-WBRT	
PCI only	3 (13)
Oligometastatic brain disease^∗^	21 (87)
Radiotherapy modality	
LINAC-based	19 (79)
Tomotherapy	5 (21)
Chemotherapy after HA-WBRT	
Yes	13 (54)
No	11 (46)
EGFR-TKI targeted therapy after HA-WBRT	
Yes	8 (33)
No	16 (67)

∗ According to our predefined criteria, oligometastatic brain disease indicates that the number of brain metastatic lesions is ≤4 on magnetic resonance imaging. EGFR-TKI = epidermal growth factor receptor tyrosine kinase inhibitor, HA-WBRT = hippocampal-avoidance whole brain radiotherapy-brain radiotherapy, LINAC = linear accelerator, NSCLC = non-small cell lung cancer, PCI = prophylactic cranial irradiation, SCLC = small cell lung cancer.

Dosimetry evaluation of organs showed that the volume of the hippocampi ranged from 3.6 to 11.5 mL (median, 4.9 mL), that of HA ranged from 15.2 to 76.9 mL (median, 34.2 mL), and that of whole-brain parenchyma ranged from 1209 to 1614 mL (median, 1370 mL). In the HA-WBRT planning dose, 22 patients (92%) received ≥30 Gy, whereas 2 patients received <30 Gy for HA-PCI. Thirteen patients (54%) received a boost dose for gross metastatic diseases, ranging from 7.5 to 20 Gy (median, 10 Gy). The coverage of (V_30Gy_ PTV) ranged from 69% to 96%, with a median of 84%, which deviated from the recommended protocol's criterion of 95%. The median PTV volume (≥25 Gy) ranged from 85% to 100% (median, 95%), which also deviated from the protocol's criterion of D_98%_ PTV ≥ 25 Gy. However, only 1 patient's plan deviated from the recommended criterion of D_2%_ PTV ≤37.5 Gy. The maximal dose delivered to hippocampus ranged from 9.2 to 25.8 Gy, with a median of 13.1 Gy.

**Figure 2:**
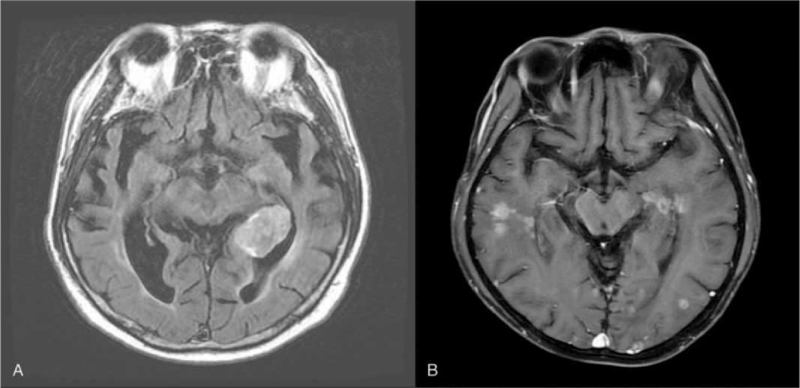
Intracranial failure in 2 patients with perihippocampal recurrences after HA-WBRT. Patient A was a 53-year-old man with limited-stage small cell lung cancer who developed a single perihippocampal failure 13 months after HA-prophylactic cranial irradiation. Patient B was a 41-year-old man with lung adenocarcinoma who developed diffuse brain failure 22 months after HA-WBRT. HA = hippocampal-avoidance, HA-WBRT = hippocampal-avoidance whole-brain radiotherapy.

During clinical follow-up, 14 patients developed progressive systemic metastatic lesions outside the brain. Only 3 patients developed intracranial failure after HA-WBRT. Two patients had intracranial failure in the perihippocampal region 13 and 22 months after HA-WBRT (Fig. 2A and B). Patient A had SCLC, clinical stage IIIB (T4N2M0), diagnosed in August 2017. He received 3 cycles of chemotherapy and showed partial response following treatment, then received concurrent chemoradiotherapy for a lung tumor, along with HA-PCI (27 Gy in 15 fractions). He developed a single perihippocampal failure 13 months after HA-PCI. The volumes of the hippocampus, HA region, and whole-brain parenchyma were 4.2, 31.9, and 1599 mL, respectively. The percentage of brain volume occupied by the HA region was 2.5%. The minimum and maximum doses administered to the hippocampus were 6.8 and 10.7 Gy, respectively (Table 2). Patient B had lung adenocarcinoma, clinical stage IV (T1bN0M1b) diagnosed in June 2015, who first underwent targeted therapy with Gefitinib, an epidermal growth factor receptor tyrosine kinase inhibitor. Two brain metastatic lesions (bilateral frontal regions) occurred 10 months following therapy. HA-WBRT was performed, with a boost dose to the gross metastatic lesions (35 Gy in 14 fractions). The volumes of the hippocampus, HA region, and whole-brain parenchyma were 3.1, 40.7, and 1452 mL, respectively (Table 2). The percentage of brain volume occupied by the HA region was 2.2%. The minimum and maximum doses administered to the hippocampus were 5.4 and 10.6 Gy, respectively. The patient continued anticancer therapy with various regimens, and developed diffuse intracranial failure, including perihippocampal recurrence 22 months after HA-WBRT. The mean irradiated doses of both hippocampal regions were 10 Gy, and the minimum doses were 6.7 Gy. The third intracranial failure was in the parietal brain parenchyma outside the region of the bilateral hippocampus.

**Table 2:** Summary of dosimetry parameters in HA-WBRT.

Characteristics	Patients (N = 24) n (%)	Patient A	Patient B
Hippocampus volume, mL, median (range)	4.9 (3.6–11.5)	4.2	3.1
HA volume^∗^, mL, median (range)	34.2 (15.2–76.9)	31.9	40.7
Brain volume, mL, median (range)	1370 (1205–1614)	1599	1452
HA regions/brain volume (%) (range)	2.4% (1.2–5.4)	2.5	2.2
HA-WBRT planning dose		27 Gy	35 Gy
≥30 Gy	22 (92)		
<30 Gy	2 (8)		
Boost to metastatic brain diseases			
Yes	13 (54)	No	Yes
No	11 (46)		
Boost dose, Gy, median (range)	10 (7.5–20)	−	−
V_30 Gy_ PTV coverage,^†^ median (range) (%)	84 (69–95.5)	85	69
V_25 Gy_ PTV, %, ^‡^ median (range)	95 (85–100)	93	85
D_max_ of hippocampus, Gy,^§^ median (range)	13.1 (9.2–25.8)	10.7	10.6

∗ HA regions are generated by three-dimensionally expanding the hippocampal contours by 5 mm volumetrically.

† Percentage of PTV brain volume receiving a dose of >30 Gy.

‡ Volume of PTV receiving a dose of >25 Gy.

§ Maximum dose administered to the hippocampi. HA = hippocampal-avoidance, HA-WBRT = hippocampal-avoidance whole-brain radiotherapy, PTV = planning target volume.

To explore whether a deviation was related to intracranial failure, we evaluated the dosimetry parameters in HA-WBT planning for our cohort (Table 2). Compared with the recommended protocol's criterion of 95% PTV receives >30 Gy coverage, patients A and B, who experienced intracranial failure, were under-covered (86% and 69% of PTV). For the recommended criterion 98% PTV > 25 Gy, patients A and B received 93% and 85%, respectively.

## 4 Discussion

The appropriate use of WBRT can provide rapid attenuation of neurologic symptoms, improve quality of life, and is especially beneficial in patients whose brain metastases are surgically inaccessible or when the patient is not fit for neurosurgery.^[1–3]^ To minimize potential long-term morbidity following WBRT, patients with limited intracranial disease are recommended focal therapeutic options, such as neurosurgical resection or stereotactic radiosurgery, to prevent long-term risks of serious cognitive deterioration and decline in function of learning and recall.^[4–5,21–22]^ With the efficacy of modern targeted therapies, patients with brain metastases survive longer, even for years following WBRT.^[3]^ Although control of metastatic brain lesions by WBRT might be the most urgent factor in stabilizing neurocognitive functions, neurological sequelae do occur with longer survival.^[5,7]^

Clinical studies have demonstrated that modest doses of radiation cause an early and significant decline in neurogenesis in the dentate gyrus of the hippocampus; this is associated with impaired recall and suppression of new memory formation. The HA-WBRT technique, to preserve neurocognitive function, is a feasible radiotherapy option in limited intracranial metastasis without hippocampal involvement.^[7–10]^ RTOG 0933 was a single-arm prospective phase II trial to confirm HA-WBRT's effectiveness in preserving neurocognitive function.^[8]^ The phase II trials confirmed that there were no differences in intracranial progression and overall survival, and that HA-WBRT could be recommended as a standard of care for brain metastatic patients with good performance status and no metastasis in the HA region.^[9,10]^ HA-WBRT will find widespread acceptance following its rigorous validation in prospective clinical trials that examine its ability to confer neurocognitive protection while preventing tumor progression.

By reducing the dose delivered to hippocampal areas to below the therapeutic level, the risk of hippocampal progression is potentially increased. In metastatic brain disease, it is presumed that the entire brain is seeded with micrometastatic disease, and that metastases are equally distributed within the brain, even when only limited intracranial lesions are detected in imaging studies. Gondi et al reviewed 371 metastatic brain patients and found that only 8.6% of cranial metastases were within 5 mm of the hippocampus; none were within the hippocampus.^[6]^ Another larger study investigated 632 patients with 6064 metastatic brain lesions. Only 4.1% of the patients developed hippocampal metastases, whereas 5.5% developed perihippocampal metastases.^[23]^ Several studies reported low rates of perihippocampal metastases with around 1% to 8.8% estimated incidence, even in brain metastasis-prone lung and breast cancer patients.^[6,11,23–26]^ Thus, it is suggested that the hippocampus and perihippocampal area are uncommon sites of brain metastasis.

Perihippocampal failure is an uncommon event following HA-WBRT. More evidence of this phenomenon must be accumulated from clinical observations and trials. In our study, Patient A, with SCLC, developed a single cranial failure in the left perihippocampal region after receiving HA-PCI. A similar case report described a patient with limited-stage SCLC who received HA-PCI and subsequently developed a solitary brain metastasis in the HA region.^[19]^ Cho et al reported 48 patients with SCLC who underwent HA-PCI; 2 developed perihippocampal recurrence. It was suggested that HA-PCI may be associated with an increased risk of intracranial failure, whereas HA-PCI did not impair disease control or survival.^[20]^ Redmond et al reported 20 patients with SCLC who received HA-PCI; 2 patients developed metastases in the under-dosed region, 1 on the dentate gyrus and 1 around the HA region.^[27]^ A phase 3 randomized trial of PCI with or without HA in SCLC concluded no difference was observed between the arms in the incidence of brain metastasis or in overall survival rates, neither in patients with stages I to III or stage IV disease. No patient with HA-WBRT developed a single metastasis within the hippocampus or under-dosed region, however, and they did find 5 patients with multiple brain metastases in the HA-PCI arm including metastasis in the under-dosed region.^[28]^ In the newly revised guidelines for limited-stage SCLC, HA-PCI is recommended as a new principle of radiation therapy to improve the preservation of cognition.^[29–30]^ However, HA-PCI may be associated with a risk of perihippocampal recurrence. Clinical decision-makers make a trade-off between the risk of perihippocampal failure and the preservation of neurocognitive function.

In our study, Patient B had lung adenocarcinoma, and relapsed with diffuse intracranial progression, including the perihippocampal region, and systemic failure; this might be attributable to uncontrolled lung disease refractory to targeted and systemic therapy. Gondi et al reported 113 patients, most of whom had lung cancer, who underwent HA-WBRT. Sixty-seven patients developed intracranial progression, which was near the HA area in only 3 patients (4.5%).^[8]^

HA-WBRT should be accepted following its rigorous validation in prospective clinical trials that examine its ability to confer neurocognitive protection while retaining its efficacy in preventing subsequent brain failure. RTOG 0933 recommended stringent dose criteria which required a high level of dose homogeneity and precise radiation delivery. We evaluated the dosimetry parameters of HA-WBRT in our cohort. Perihippocampal failure occurred in 2 of 24 patients (8%) in our cohort. It is difficult to all attribute perihippocampal failures to inadequate PTV coverage of radiation dose. Our knowledge of the dose–toxicity relationship for the hippocampus is still evolving; however, it is known that a higher radiation dose leads to more neural stem cell death. However, with HA-WBRT, inadequate plan deviations from the RTOG 0933 criteria might be common in clinical practice. Rong et al reported HA-WBRT treatment times of 15 minutes for IMRT and 18 minutes for Tomotherapy.^[18]^ Our previous study of LINAC-based HA-WBRT reported average delivery times of 289 ± 19.4 seconds (coplanar) and 372 ± 19 seconds (noncoplanar).^[14]^

In Taiwan, brain metastasis cancer patients are funded by the National Health Insurance system for palliative therapy only. Thus, it is difficult to use HA-WBRT widely in routine radiotherapy; the significant cost of optimizing hippocampal dose reduction and PTV coverage is not fully reimbursed. Therefore, a dilemma exists in balancing clinical workload with the time-consuming planning necessary for the fulfillment of recommended criteria.

The prognosis of patients with brain metastasis was poor before the 21^st^ century; the overall survival of treated patients was approximately 4 to 6 months.^[3]^ In recent decades, advancements in targeted therapy and immunotherapy have obtained better outcomes. However, survival still varies in brain metastases cancer patients, influenced by many factors. Yamamoto reported that the median overall survival of patients with solitary brain metastasis was 13.8 months; for those with multiple brain lesions, 10.8 months.^[22]^ Although our study excluded patients with solitary brain metastasis, we found a median survival of 12 months following HA-WBRT, which is comparable to other published results of WBRT.^[1–3]^

This study has some limitations. First, because of the study's retrospective nature and small sample size, it was possible only to imply causality for perihippocampal failure in HA-WBRT. Second, we had not evaluated neurocognitive test before or after HA-WBRT in our cohort. Various systemic therapies can influence clinical outcomes in cancer patients with brain metastasis. Due to the aggressiveness of the tumors might have been different from the actual tumor volume, cancer stage, or known prognostic markers including Ki-67 index, or tumor growth proteins which can determine aggressiveness. This is the true limitations of this study. We explored the clinical manifestation of cancer patients with brain metastases following HA-WBRT, and reported unusual intracranial failure in the HA region. Although HA-WBRT is accepted as a standard therapy for patients with limited brain metastases, perihippocampal failure might be attributable to under-dosing of radiation in planning coverage partially or be caused by aggressiveness of cancer per se. Further research on this topic is encouraged.

## Author contributions

**Conceptualization:** Sheng-Yow Ho.

**Data curation:** Li-Tsun Shieh, Sung-Wei Lee, Chia-Chun Chen, Yi-Chia Ho, Yu-Wen Wang, Sheng-Yow Ho.

**Formal analysis:** Li-Tsun Shieh.

**Funding acquisition:** Li-Tsun Shieh.

**Investigation:** Sheng-Yow Ho.

**Methodology:** Sheng-Yow Ho.

**Project administration:** Sheng-Yow Ho.

**Resources:** Sung-Wei Lee, Chia-Chun Chen, Yu-Wen Wang, Sheng-Yow Ho.

**Supervision:** Sheng-Yow Ho.

**Validation:** Sheng-Yow Ho.

**Writing – original draft:** Li-Tsun Shieh.

**Writing – review & editing:** Sheng-Yow Ho.
